# The Research of Acupuncture Effective Biomolecules: Retrospect and Prospect

**DOI:** 10.1155/2013/608026

**Published:** 2013-11-02

**Authors:** Yu Wang, Lei-Miao Yin, Yu-Dong Xu, Yan-Yan Lui, Jun Ran, Yong-Qing Yang

**Affiliations:** Molecular Biology Laboratory, Shanghai Research Institute of Acupuncture and Meridian, Yue Yang Hospital, Shanghai University of Traditional Chinese Medicine, Shanghai 200030, China

## Abstract

Acupuncture is an effective, safe and convenient therapy that has been applied for 2,500 years. The acupuncture researches have obtained significant improvement with the technical support of the life sciences and the studies of acupuncture have in turn accelerated the development of biomedical science. The effects of acupuncture influence important physiopathologic and biological activities, including gene expression, protein-protein interactions, and other biological processes. Cerebrospinal fluid, serum, organs, and tissues are reported to be carriers of the biomolecules of the effects of acupuncture. The paper summarized the progress of acupuncture effective biomolecules researches and found that biomolecules play important roles in the mechanism of acupuncture. With the development of omics technologies and translational medicine, the acupuncture research will meet both opportunities and challenges.

## 1. Introduction

Acupuncture is one of the key components of traditional Chinese medicine involving the insertion of solid filiform needles into the skin at specific points on the body to achieve therapeutic effects, acupuncture is considered to be the ancient Chinese art of healing [1, 2]. Using ancient scientific principles, acupuncture treats illnesses by bringing a person's body into harmony and regulating the balance of yin and yang. Since the Warring States period (475 BC–221 BC), acupuncture has been utilized for more than 2,500 years in China [3]. This technique spread throughout the Far East and Europe, then to America during the 19th century [4].

There have been two waves of development of acupuncture research since 1950s [5], when acupuncture was used as a technique to induce analgesic effect in the place of anesthetics during surgical procedures. The first wave was started in the late 1950s [6]. The Chinese government began to invest in research on acupuncture, and this nonconventional practice raised the interest of not only medical professionals over the world, but also basic researchers who would like to explore the possible mechanisms. What really launched acupuncture in the West, however, was a report in the New York Times in 1971 [7]. The report inspired a rush of American medical doctors to China to investigate acupuncture analgesia. There was a marked increase in acupuncture research in the 1990s. In terms of the increase in the number of SCI-Expanded journal papers, acupuncture literature can be divided into two phases. The first phase dated from 1973 to 1997, and during these years, the number of articles fluctuated to plateau at an average of 85 papers per year. The number of articles showed a dramatic 40% increase in 1998 and continued to increase [5]. This growth might be encouraged by two events. The first one was the “NIH Consensus Development Conference on Acupuncture” held in 1997 in Bethesda, MD. The second event was that the National Center for Complementary and Alternative Medicine (NCCAM) was inaugurated under the USA NIH in 1998 [5].

In recent years, the researches of Chinese medicine, specifically acupuncture, have shown significant improvement with technical supports of the life sciences. Simultaneously studies in acupuncture have in turn accelerated the development of medicine as well as understanding of biomedical science. For example, the achievements of fifty years of acupuncture anesthesia research and application have enriched the gate control theory of pain and clarified the underlying mechanism of endorphin release. In the last half of century scientific research on acupuncture, coupled with advances in knowledge about pain control mechanisms, has yielded facts sufficient to develop acupuncture analgesia [8]. The principle research has been developing for nearly half century, during which acupuncture anesthesia has played an important role and made remarkable contribution to research hypotheses and methods. The research of acupuncture is not only a method but also the source of the development of concept, cognition, and methodology. A systematic review outlining 50 years of principal research of acupuncture in China and summarizing the basic regulation and characteristics of acupuncture may play a guiding role in the future development of this discipline.

Biological systems are composed of two factors of information: genes and networks of regulatory interactions. This information is hierarchical in nature: DNA → mRNA → protein → protein interactions → informational pathways → informational networks → cells → tissues or networks of cells → an organism → populations → ecologies [9]. Other macromolecules and small molecules also participate in these information hierarchies [9]. The effects of acupuncture are through the influences on physiological and pathological processes after the stimulation on acupoints. The research on biomolecules of acupuncture goes a long way towards explaining the biological processes from gene expression and protein functional expression as well as the information reaction sequence and rule of protein interaction.

The acupuncture effective biomolecules are biological molecules which have similar acupuncture effect and were produced by a living organism response to acupuncture, including macromolecules and small molecules, such as proteins, polysaccharides, lipids, nucleic acids, and primary and secondary metabolites. It is reported that the cerebrospinal fluid, serum, organs, and tissues of the acupoint are the carriers of biomolecules of acupuncture effects, which contain various acupuncture effective biomolecules and have acupuncture-like effect added into experimental system *in vivo* or *in vitro*. This review aims to summarize the progress of acupuncture effective biomolecules researches, which provides clues and guides for the acupuncture development.

## 2. Acupuncture Effective Biomolecules in the Cerebrospinal Fluid

The effect of acupuncture analgesia for surgery is based on chemical mediations. The transfer of the cerebrospinal fluid (CSF) of rabbit under acupuncture analgesia to the third ventricle of a naive recipient animal produced an analgesic effect in the second rabbit. The results showed that transmitters in the CSF were responsible for the analgesic effect [10]. Li et al. showed that the classical neurotransmitter serotonin was an important mediator of acupuncture analgesia [11]. Han et al. [12, 13] used the antibody injection technique to show that enkephalins and beta-endorphin are mediators for acupuncture analgesia in the brain. Dynorphins were effective in the spinal cord but not in the brain [14]. Important correlations of the endorphins in acupuncture analgesia hypothesis were found in the report of Sjolund et al. [15], which showed that endorphins were increased in the CSF after electroacupuncture stimulation.

Different frequencies of stimulation can affect the release of different neuropeptides. Han et al. [16] showed with serial samples of CSF from human volunteers that different types of neuropeptides can be released in the CNS by simply changing the frequency of electrical stimulation without moving the position of the needle. Low frequency (2 Hz) electroacupuncture increases the content of beta-endorphin and met-enkephalin in the CSF, whereas high frequency (100 Hz) accelerated the release of dynorphin. This scientific evidence of frequency-specific effects that are wide spread throughout the CNS was different from the symptom-specific metaphysical theories of specific acupoint needle stimulation. However, stimulation of different points representing different neurotomes can also produce action on those body structures innervated by the neurotomes.

The above-mentioned work was the milestone of acupuncture research. The National Institute of Health consensus conference in 1997 recognized acupuncture (and by extension Traditional Chinese Medicine) as a legitimate branch of scientific medicine. Acupuncture has received an enormous boost in the last few years and seems destined to be accepted and incorporated into Western medicine. Research on acupuncture analgesia has shown a substantial basis for acupuncture and promoted the development of neurophysiology.

## 3. Acupuncture Effective Biomolecules in Serum

Serum includes all of the proteins not used in blood clotting (coagulation) and all the electrolytes, antibodies, antigens, hormones, and any exogenous substances (e.g., drugs and microorganisms). The serum of an animal is used to provide immunity to a pathogen or toxin by inoculation or as a diagnostic agent [17]. With biological activity, as carrier of acupuncture effective components, serum has received a great deal of attention during the past twenty years in China.

In the regular experiment, serum was considered to be an ordinary sample. However, serum was now recognized as carrier of acupuncture effective biomolecules. Regardless of the source of human or animal, sera that prior to or after acupuncture treatment were added into an *in vitro* reaction system as an effect substance. Through contacting with target objectives biomolecules of acupuncture effects, serum effects can be observed to evaluate the function of acupuncture and moxibustion [18]. The acupuncture effect is closely associated with the induced specific proteins by acupuncture and moxibustion. This will become an epoch-making discovery when “the needling or moxibustion can induce specific proteins” and can be proved as a universal phenomenon [18].

Serum after acupuncture (SA) shows biological activity similar to acupuncture* in vivo *and *in vitro*. In the *in vivo* experimental system, the SA was injected into an experimental animal. After the intravenous injection of SA, SA can decreased the eosinophil counts in the peripheral blood [19]. To observe the SA effects on a cell *in vitro*, a cell was cultured within a culture media containing SA. A different research work showed that SA can reduce the number of osteoclasts *in vitro* [20], decrease the Ca^2+^ level in cultured myocardial cells [21], decrease the Ca^2+^ level in cultured cells of the cerebral cortex [22, 23], and promote the growth of a tumor-infiltration lymphocyte both in the aspects of proliferation and phenotypes [24].

The research on SA *in vivo *and *in vitro* provides direct evidence of biomolecules in SA. The acupuncture serum samples were separated by gel filtration into three segments according to molecular weight [25], and each segments could decreased the eosinophil counts in the peripheral blood. The effective components of acupuncture serum from asthmatic rats treated by acupuncture for eosinophils were not single component, and acupuncture stimulation may produce many types of components of antiasthma [19]. In further research, two-dimensional protein electrophoresis was employed to analyze the differential proteins in the serum with antiasthma activity of acupuncture, and differential proteins such as cyclophilin A and zinc finger protein 91 were identified with mass spectrometry. There were multitarget effects regulating the whole body in acupuncture treating asthma relating to immunoregulation, gene expression, and proteins synthesization. The effects of acupuncture on the response of proteins warrant further research [26, 27].

## 4. Acupuncture Effective Biomolecules in Organs

Clinical observation and principal research on acupuncture focus on the adjustment of the zang-fu organ and have shown that the adjustment by acupuncture relied largely on the neuroendocrine-immune network, which also provide new evidences for the acupuncture effective components in different organs. 

Four SAGE libraries of the lungs of the control rats (CK), asthmatic rats (AS), asthmatic rats treated by acupuncture (ASAC), and control rats treated by acupuncture (CKAC) were established and bioinformatics analyses were conducted. The study found that the gene expression profile of the AS and ASAC was more similar than that of the other groups by the hierarchical dendrogram; 21 specific genes regulated by acupuncture in the asthmatic model, such as the S100 calcium binding protein A9 (S100A9), metallothionein-2 (MT-2), and the dual specificity protein phosphatase 1 (Dusp1) were found by Venn graph; three key gene categories, such as “immune response,” “response to steroid hormone stimulus,” and “homeostatic process,” were closely associated with acupuncture treatment for asthma via DAVID functional analysis; DAG analysis suggested that acupuncture was a biological process which regulated the genesis of endocorticosteroids and inhibited immune response in asthma treatment; the KEGG pathways indicated that the genesis and regulation of the hormone and immune response were involved in acupuncture treatment for asthma [28]. Using two-dimensional gel electrophoresis (2DE) and mass spectrometry, pulmonary proteins such as S100A8, S100A11, and the Clara cell 10-kDa protein (CC10) were found, which could be used to identify new drug candidates for the prophylaxis and treatment of asthma [29]. 

Several key effective molecules of acupuncture were selected for validation of their function. As a member of the S100 family, the S100A9 protein, which originate from lung, elicits dose-dependent antiasthmatic effects and may provide further insight into the treatment of asthma [30]. The CC10 protein that is secreted by the nonciliated, nonmucous, secretary epithelial Clara cells of the pulmonary airways showed that it could inhibit the proliferation of airway smooth muscle cells and migration induced by platelet-derived growth factor (PDGF), and this suppressive effect might be associated with the inhibition of cyclin D1 expression [31].

Pulmonary functions changes were closely related to the rectal resting pressure in the rat model of asthma and constipation, and the lung homogenate could significantly contract the large intestine muscle strip [32]. This relationship could be effectively regulated by acupuncture, and the phenomenon suggested that there were effective biomolecules in the lung homogenate.

## 5. Acupuncture Effective Biomolecules in Acupoints Tissues

Researchers have investigated the specific structure of the acupoint tissues, but there has been limited progress without convincing results. In the last 20 years, research on the local molecular mechanism of acupuncture has progressed gradually. Preliminary research suggested that histamine and adenosine were effective biomolecules of acupuncture information generated locally at acupoint.

During the needling manipulation process, the needle is being grasped by connective tissue as a result of collagen and elastic fibers winding and tightening around the needle, delivering a cellular signal conducted along the pathway of channels (meridians) and leading to downstream effects that activate certain cellular pathways and facilitate healing [33]. Collagen fibers play an important role in acupuncture-induced analgesia, and they participate in signal transmission and transform processes [34]. The analgesic effect was more pronounced after stimulation of the Zusanli (ST36) point than after stimulation of a sham point near the true acupuncture point. The density of mast cells from the Zusanli (ST36) point of rats was higher than that from a nearby sham point. In addition, acupuncture resulted in a remarkable increase in degranulation of mast cells. Disodium cromoglycate (DSCG) is in the mast cell stabilizers, caused a concentration-dependent inhibition of histamine release [35, 36]. Pretreatment of the acupuncture point with disodium cromoglycate (DSCG) not only counteracted the phenomenon of degranulation but also reduced the analgesic effect of acupuncture. Experiments on inhibition of the degranulation of mast cells in tissue from acupuncture points demonstrate the possible role of mast cells in the effects of acupuncture [37].

The mast cell densities were higher in the acupoints than in the nonacupoints. Effective nerve conduction signaling in manual acupuncture (MA) analgesia was generated after the degranulation of mast cells in the process of acupoints activation or needle sense, which was the direct cause of generation of the nerve signals [38]. There was a positive correlation between the mast cell degranulation rate and the analgesic effects [39]. After activation, mast cells express histamine, leukotrienes, and prostanoids, as well as proteases, and many cytokines and chemokines [40, 41]. These mediators were pivotal to the genesis of an inflammatory response [42]. Transmitters released by mast calls can increase vascular permeability and induce local edema and mild authigenic inflammatory response [43]. On one hand, this possibly promotes aggregation of mast cells in focal and other places towards acupoints via induced adhesion molecules and chemotactic factors; on the other hand, it can activate immune system, and expand local acupuncture effect to the entire body [44]. Acupuncture has a significant analgesia and enhances the degranulation of mast apparently cells, which was weakened by injection of disodium cromoglycate (DSC) in the acupoint area, suggesting an important role of mast cells in acupuncture-induced analgesia [45].

Adenosine is a neuromodulator with antinociceptive properties [46]. ATP is released in response to mechanical and electrical stimulation or heat. Once released, ATP acts as a transmitter that binds to the purinergic receptors [47, 48], and it cannot be transported back into a cell but was rapidly degraded to adenosine by several ectonucleotidases before reuptake [48]. Thus, adenosine acted as an analgesic agent that suppresses pain through Gi-coupled A1-adenosine receptors [49, 50]. A researcher collected samples of interstitial fluid by a microdialysis probe implanted in the tibialis anterior muscle/subcutis of adult mice at a distance of 0.4–0.6 mm from the Zusanli (ST36) point, and the adenine nucleotides and adenosine were quantified using high-performance liquid chromatography (HPLC) before, during, and after acupuncture. Adenosine was released during acupuncture and its antinociceptive actions required the adenosine A1 receptor expression. Direct injection of an adenosine A1 receptor agonist replicated the analgesic effect of acupuncture. Inhibition of the enzymes involved in adenosine degradation potentiated the acupuncture-elicited increase in adenosine, as well as its antinociceptive effect. The observations indicate that adenosine mediates the effects of acupuncture and interfering with adenosine metabolism may prolong the clinical benefit of acupuncture [51].

Further research was conducted with human subjects. The interstitial adenosine concentration increased significantly during acupuncture and remained elevated for 30 minutes after acupuncture. Acupuncture-mediated adenosine release was not observed if acupuncture was not administered in the Zusanli (ST36) point or if the acupuncture needle was inserted, but not rotated. The study strengthened the role of adenosine in acupuncture-mediated antinociception by directly providing such evidence in humans [52]. The research presents further evidence of the role of adenosine in acupuncture-mediated antinociception by demonstrating that local adenosine concentrations increase in the acupoint in human subjects receiving traditional acupuncture.

## 6. Perspective

Acupuncture is the pilot subject to increase global acceptance of Chinese medicine. This technique is accepted by the scientific community because it is an effective therapy, the biological effect of acupuncture is unique, and it has biological significance for the research of the human biology. Previous research on acupuncture anesthesia principle research played an important role by establishing the basis and methodology of acupuncture research, which has made an important scientific contribution. The research investigating acupuncture anesthesia and acupuncture analgesia promoted the knowledge of pain physiology.

Since 1998, global acupuncture research has developed rapidly [5]. In the beginning of the 21st century, the acupuncture research has also passed on to the postgenome era [53]. The effects of acupuncture comprise a complicated biological process, and many biological molecules are involved. Systems biology techniques, such as functional genomics and proteomics, are involved in an increasing number of applications in the field of acupuncture research. With the development of biological technology such as high-throughput omics technology (genomics, transcriptomics, proteomics, metabolomics, and beyond) [54], the fundamental biological processes of acupuncture can be studied by applying the full range of omics technologies to reveal the mechanism of the effect of acupuncture.

The effects of acupuncture include non-specific effects and specific effects and acupuncture effective biomolecules can be either nonspecific effective biomolecules or specific effective biomolecules. Endogenous opioid peptides (enkephalin, dynorphin, endorphins, and orphanin) and purine (adenosine) were significant but non-specific effective biomolecules of acupuncture anesthesia and acupuncture analgesia. These non-specific effective biomolecules are produced by the acupuncture and are not based on a specific body state. Biomolecules originate from specific organs of the disease model, such as S100A9 and CC10, and represented the specific effect of acupuncture. Additional specific effective biomolecules could be discovered by more research into the different types of diseases.

Translational medicine is a relatively young area of biomedicine, and it has developed rapidly and become more interdisciplinary in the past 10 years [55], which is becoming ever-more interdisciplinary. The combination of translational medicine and acupuncture effectiveness and target drug discovery based on acupuncture effect biomolecules will promote the application of acupuncture. These combined processes will also promote the development of biological medicine.
